# Report of Surgical Cases

**Published:** 1864

**Authors:** George K. Ammerman

**Affiliations:** Attending Surgeon to the Chicago City Hospital


ARTICLE XXXII.

### REPORT OF SURGICAL CASES.

By GEORGE K. AMMERMAN, M.D., Attending Surgeon to the Chicago City Hospital.

CASE I.—*Removal of Tumor from the Mouth—Successful.*

Mrs. B. aged about 35, came to Chicago in May, 1860, to obtain advice relative to a swelling, situated immediately under the tongue. She stated that, about six months previously, she first noticed the growth as a small, hard, painful swelling, about the size of a bean. It came without any assignable cause. She consulted a physician who pronounced it an abscess and opened it, but without any good effect. She next consulted a surgeon, who introduced a seton through it. This was left in three days, when her sufferings became so severe that she could not possibly endure it. In the meantime, the swelling greatly increased in size, and constitutional symptoms set in.

At this period, May, 1860, I was consulted; I found her greatly reduced in strength, anemic, and with some febrile action. The tumor occupied the whole of the sublingual region, extending from side to side, and raising the tongue up into the roof of the mouth; it was hard, solid, and unyielding; the sublingual veins enlarged and tortuous; it was marked on its upper surface by a deep cicatrix, and over that part seemed more dense; it interfered with articulation and deglutition, and kept up a constant flow of saliva. It was at this time free from pain.

I advised her to return home, use iron, exercise in the open air, good diet, and await further developments before resorting to an operation for its removal. In October, 1863, two years afterwards, she returned to this city. During this time her general health had greatly improved; she was still rather anemic, but otherwise seemed in perfect health. The tumor presented nearly the same appearance as at the previous examination; it had very slightly increased in size; seemed somewhat firmer, and to extend further back into the throat. I advised its removal, and with the assistance of Dr. C. G. SMITH, of this city, excised the whole of it, on Thursday, the 8th of October.

Its removal was attended with some dfficulty, as no anesthetic could be used, and great care required to avoid the larger vessels; the hemorrhage was slight, and altogether, she bore the operation in a most remarkable manner. The after treatment was simple; and in a few days she left the city.

I have recently been informed that her health remains good, and there are no indications of a return growth. The tumor, after its removal, was oblong in shape, as large as the largest sized hen's egg; no microscopical examination was made; to the eye it seemed to be composed exclusively of parallel fibres easily separated into layers; it was contained in a firm cyst, to which it was closely adherent.

*Remarks.*—The interest and importance of the above case, depends solely upon its correct diagnosis. It is quite evident, from the history, that its nature was not entirely apprehended by those gentlemen who were first consulted in regard to it; and, to be altogether candid, I must confess, that when I first saw the case, I was fully convinced of its malignancy, and advised a course of treatment in accordance therewith. The symptoms, at that time, constitutional and local, were all, in my view, in favor of such an hypothesis. At my second examination, made two years afterwards, the condition had so much changed that the diagnosis was easy enough. It had then existed for a long time; had been of slow growth; not very painful; had not involved surrounding parts, and had not produced any constitutional disturbance, all of which clearly indicated its simple, benign character. Its nature decided, the treatment was plain.

There is no question as to the utility of an operation in cases of simple, benign growths. For the most part they are entirely beyond the reach of medicines, and the only question to be settled is, whether we shall extirpate them, or let them alone, and this should always be settled by the patient, without active interference on the part of the surgeon.

CASE II.—*Amputation of the Thigh for Railroad Injury—Erysipelas—Recovery.*

W. T. B. a strong, healthy man, was run over by the cars, on Clark Street, on the morning of June 16, 1863. I saw him about two hours after the accident occurred; he was lying on the floor, in a small tenement house, screaming with pain; his right leg was crushed into a pulpy mass from the ankle to the knee; the foot and knee joint were untouched; there was no shock. He was placed on the bed, and with the assistance of Drs. ROSS and AVERY, the limb amputated in the lower third of thigh, by anterior and posterior flap method. Simple dressings were applied, and a full dose of opium given for the night.

June 17th, pulse 120—reaction high; ordered cold water to be constantly applied, by dropping, to the stump; internally, fever mixture, containing digitalis and opium, in full doses at night.

June 18th, no change, same treatment continued.

June 21st, erysipelas developed last night in outer side of wound, and extends to hip; fever high, with great pain and restlessness; ordered quinine and iron, full doses, beef tea, brandy, and opium.

June 24th, erysipelas subsiding.

July 30th, since last note patient has been very low; a large abscess formed on the outer side of the thigh, which discharged profusely, with hectic and night sweats, requiring tonics and supports in full quantities.

September 22d, ligatures came away to-day, three months after the operation.

*Remarks.*—Primary amputation of the thigh, for railroad injury, is among the most fatal operations in surgery; and, although nothing of special interest attends the above case, the result renders it worthy of publication. The erysipelas was severe, resulting in the formation of an extensive abscess and profuse suppuration. The treatment of the case, throughout, was on general principles; tonics and support being the main reliance. At one time, the sulphites were tried but without producing the slightest effect.

CASE III.—*Lithotomy—Secondary Hemorrhage—Recovery.* James Burns, aged 9, of this city, began to suffer from pain in the back, with frequent and painful micturition, in November, 1863. In December, his parents consulted Drs. MARGUERAT and HEYDOCK—Dr. M. carefully examined the case, detected stone in the bladder, and gave some general directions regarding the treatment. On Thursday, December 3d, Drs. MARGUERAT, HEYDOCK, and myself, placed the patient under the influence of an anesthetic, and with a no. 4 steel sound, confirmed the diagnosis of Dr. M., and advised an operation for its removal, to which the friends readily assented.

On Sunday, December 6th, assisted by Drs. MARGUERAT, HEYDOCK, SMITH, HOLMES, and ROSS, the patient was fully anesthetized, the limbs held in position, and the calculus removed by lateral incision. There was very little hemorrhage attending the operation, and no ligatures were necessary. The patient was placed in bed and an anodyne given for the night.

December 7th, slept well; no pain; pulse 120; ord. demulcient drinks in large quantities; opium at night.

December 9th, considerable soreness over the bladder; pulse 120; no appetite; urine passed per urethra last night; ord. oil ricini.

December 10th, free movement of the bowels; two large blood clots expelled through wound, after which urine again passed through.

December 13th, 14th, 15th, secondary hemorrhage, at intervals since last note, in large quantity; controlled by iron, injected into the wound, and taken internally; beef tea, and support freely.

December 28th, urine passed per urethra for first since hemorrhage; wound nearly closed; everything doing well.

January 6th, passed a small calculus size of a half pea.

January 21st, wound re-opened; patient anemic, with diarrhœa and great debility; ord. cod liver oil, iron, and good diet.

February 3d, wound entirely united; patient discharged—cured.

*Remarks.*—For the notes in the above case, I am indebted to my friend, Dr. MARGUERAT, of this city, who had charge of the case throughout, and to whose careful management the result is largely due.

The case presents few points of interest, and those very apparent in its history and notes of Dr. M. The secondary hemorrhage was not of an alarming character at any time. The amount of blood lost was doubtless greatly exaggerated by the friends, but even a few ounces in his condition was a serious drawback, and considerably prolonged the cure.

